# From clicks to calories: Online-to-offline food consumption and overweight and obesity

**DOI:** 10.1371/journal.pone.0315189

**Published:** 2024-12-16

**Authors:** Yuanyi Zou, Lin Lin

**Affiliations:** Department of Urban Planning and Design, Xi’an Jiaotong–Liverpool University, Suzhou, Jiangsu, PRC China; Mahidol University, THAILAND

## Abstract

The rise in online-to-offline (O2O) take-out food consumption has become a global urban phenomenon. While links between general fast-food consumption and increased risks of overweight and obesity are well-documented, the specific relationship with O2O take-out food has not been thoroughly examined. This study aims to fill this research gap by investigating the impacts of take-out food consumption on the risks of being overweight and obese among urban residents. A cross-sectional online survey was conducted between April and July 2022. 716 adult respondents from the metropolitan areas of Shanghai and Suzhou were recruited. Being overweight and obese were measured using self-reported weight and height. Frequencies and preferences of O2O take-out food consumption behaviors were measured using the validated questions designed based on Food Frequency Questionnaires and Dietary Screeners, respectively. The International Physical Activity Questionnaire’s short form measured the physical activity level. Cronbach’s alpha and Expletory Factor Analysis were used to assess the reliability and validity of the O2O take-out food-related dietary questions. Binary logistic regression models are developed to examine associations between O2O take-out food consumption behaviors and being overweight or obese, adjusting for individual factors, physical activity level, and non-O2O take-out food consumption. The results indicated a direct correlation between frequent O2O take-out consumption and higher risks of overweight and obesity (odds ratio 1.087, 95%CI 1.068~1.106). The preference for ordering Western-style fast-food positively contributed to being overweight and obese (odds ratio 1.071, 95%CI 1.046~1.095). Conversely, a preference for Chinese-style fast food initially appeared to reduce these risks, although the association diminished when accounting for fruit and vegetable consumption. This study represents a pioneering exploration into the effects of O2O take-out food on overweight and obesity. The study results identified an association between the habitual use of O2O take-outs and an increased propensity for being overweight and obese.

## Introduction

### Background

Take-out food has become increasingly popular among urban residents globally in recent years [1]. This increasement in the preference for take-out food options has been further exacerbated by the COVID-19 pandemic, which has prompted a shift towards convenient online ordering and offline delivery services, collectively known as Online-to-Offline (O2O) platforms [2, 3]. With the proliferation of internet access and the widespread adoption of smartphones, elements such as availability, pricing, payment, marketing, and convenience have migrated online, reshaping how food is marketed, sold, and delivered [4]. Meanwhile, the offline component of O2O services remains grounded in the tangible aspects of the food consumption journey, encompassing food preparation, packaging, the actual delivery process, and the dining experience [5]. Due to the diverse menu options and wide price range, the customer base for O2O take-out food also exhibits a high level of diversity [1]. As the prevalence of O2O take-out food continues to grow, it has the potential to exert a profound influence on urban life and dietary habits [6]. Notably, the prevalence of overweight and obesity has also risen during the pandemic [7, 8]. This simultaneous rise in O2O take-out food consumption and obesity rates necessitates a critical examination of the impact of enhanced convenience on public health.

Previous studies have outlined the harmful effects of the consumption of fast food, especially in relation to gaining too much weight, obesity, and other related health problems [9–14]. Recent investigations have also found a discernible nexus between O2O take-out food consumption and the proximate accessibility of fast-food establishments [15]. Nevertheless, the extant literature remains relatively scarce in its comprehensive exploration of the specific impact that O2O take-out food has on weight-related concerns [16]. This empirical investigation seeks to enhance our understanding of O2O take-out food consumption behaviors by delving into both the frequency and individual preference aspects. It aims to elucidate the intricate connections between these factors and the propensity of urban residents to encounter issues related to excess weight and obesity.

### Literature review

Overweight and obesity are recognized as common chronic diseases worldwide, affecting both adults and children [17]. Obesity, characterized by excessive energy intake surpassing expenditure, is a multifactorial chronic condition [18, 19]. The health implications of overweight and obesity are profound. The WHO provides statistics indicating that mortality, morbidity, and disability resulting from major chronic diseases are projected to account for approximately 73% of all deaths and 60% of the global disease burden [20]. Obesity plays a significant role in the development of numerous chronic diseases, including cardiovascular disease (CVD) and type 2 diabetes, which have detrimental effects on individual and public health [21–24].

Extensive studies have delved into the links between fast-food consumption, the abundance and accessibility of fast-food outlets, and their impact on the rates of obesity and overweight [9, 10, 13, 25–37]. Meanwhile, the density and accessibility of recognized health-food providers like supermarkets, fresh fruit vendors, and vegetable markets are found to be beneficial for weight management [38–40]. While fast-food restaurants are often linked to poor nutrition and higher Body Mass Index (BMI), other dining options are perceived more favorably in terms of health. However, a comprehensive literature review reveals that eating outside the home, in general, is associated with higher energy and fat consumption, alongside a decrease in micronutrient intake [41]. This finding underscores the need for a nuanced understanding of the differences between fast-food restaurants and other types of out-of-home dining venues, as suggested by Bezerra and colleagues [42].

Moreover, the fast-food landscape is diverse, extending beyond Western-style offerings to include traditional snacks and meals in Asia, Latin America, and Africa [43]. For instance, a large segment of Singapore’s population frequently eats at hawker centers, which are vibrant, open-air complexes where a variety of local foods are available [44]. Interestingly, a comparison of Western fast food with local Asian offerings revealed little difference in energy and fat content [45], suggesting that the health effects of non-Western fast-food styles warrant further study.

The advent of digital technology has transformed the food environment. Granheim and colleagues highlighted how the digitalization of food availability, pricing, payment, and marketing has particularly affected food purchasing behaviors, ushering in a new era of online shopping and delivery services [46]. Yet, these services often promote unhealthy food options, as noted by Duthie and colleagues [47], indicating potential public health implications. While recent research has begun to profile the socio-demographic characteristics of online food order consumers [48, 49], the direct effects of this digital food environment on body weight and nutrition remain underexplored.

This empirical study aims to bridge these gaps by comprehensively examining O2O take-out food consumption behaviors, including frequency, preferences, and the variety of food types consumed. It differentiates between Western-style fast-food, non-western fast-food, and non-fast-food options to provide a clearer picture of the health implications associated with these choices.

## Method

### Study design and participants

This study specifically targeted adult urban residents residing in the metropolitan areas of Shanghai and Suzhou, located within the Yangtze River Delta region of China. The O2O industry in China has undergone rapid expansion, encompassing 421 million customers in 2019 [50]. The significance of Shanghai and Suzhou in the Yangtze River Delta region is underscored by their substantial contribution, accounting for one-third of all O2O orders in China [51]. Consequently, selecting Shanghai and Suzhou as the study’s focal points ensured access to a robust dataset, facilitating a comprehensive analysis of consumer behaviors associated with online-ordered take-out food.

The survey spanned the period from April to July 2022, involving 746 adult participants recruited through a cross-sectional online survey. The survey tool is a structured questionnaire, provided in the Mandarin Chinese version. Due to travel restrictions and the risk of COVID-19 quarantine in China in 2022, we hired a professional survey company in China, Wenjuanxing™, to distribute the questionnaire online. The company specializes in distributing questionnaires to specific demographic groups (adults aged 18 to 60) according to our requirements and has guaranteed the reliability of the samples and the quality of the responses. Therefore, this study did not place significant emphasis on the response rate. It is worth noting that in China, university students are particularly enthusiastic about ordering O2O take-out food [16]. Therefore, given that the survey company only screened for adult participants, it was expected that a significant portion of the sample would consist of university students.

Importantly, the study was ethically approved by the University Ethics Committee of Xi’an Jiaotong–Liverpool University under Proposal Number 21-02-34. The approval process involved a thorough review of the research proposal, ensuring compliance with ethical guidelines and standards. Any deviations from the approved protocol or supporting documents require prior approval from the University Ethics Committee (UEC) before implementation. Participants in the study were informed of their rights and provided with an informed consent form at the beginning of the online survey. It was made clear that participation was voluntary, with the option to withdraw at any time without explanation. Participants were also informed of their right to access and request the destruction of their provided information. Completion of the survey indicated the participant’s informed consent, and data analysis was conducted anonymously to ensure confidentiality and privacy.

**Fig 1:**
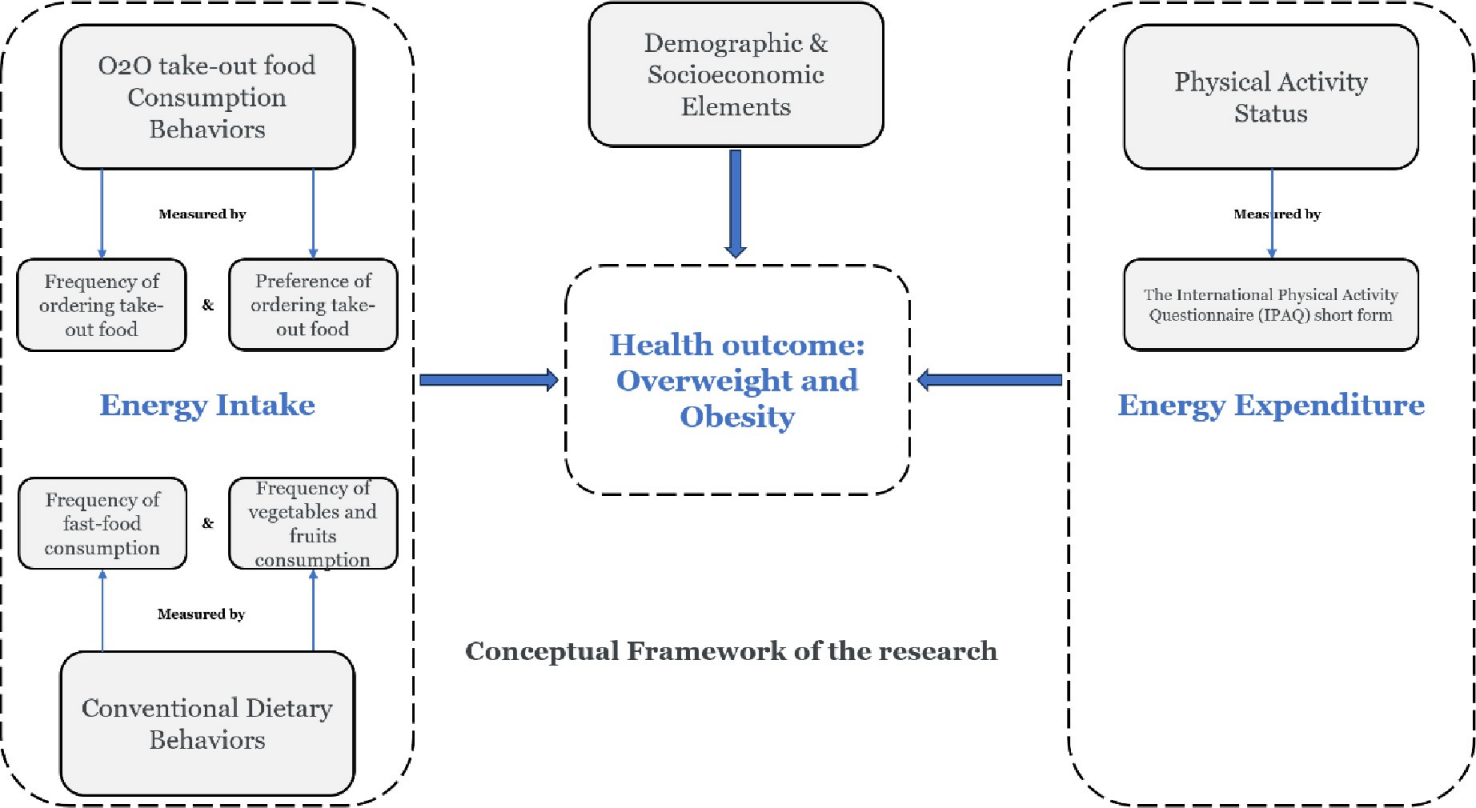
Conceptual framework of the research.

The Socio-Ecological Model on Obesity and Type-2 Diabetes, which is a multi-level and multi-sectoral framework initiated for the obesity and diabetes research [52, 53], served as the conceptual framework for the study (See Fig 1). To capture the multifaceted dynamics influencing overweight and obesity, a survey questionnaire was meticulously developed following the conceptual framework. The questionnaire comprehensively addressed various aspects, including urban customers’ energy intake and expenditure, as well as individual demographic and socioeconomic factors.

### Independent variables

The independent variables of this study are food intake behaviors including O2O and non-O2O take-out food consumption behaviors. Assessing these dietary behaviors necessitates a thorough examination of the frequency of food intake, a pivotal determinant [54–57]. To address this, survey questions pertaining to frequency were formulated based on the Food Frequency Questionnaire (FFQ)—a widely utilized tool for gauging the habitual frequency of consuming specific types of food over a specified timeframe [55, 58, 59]. Specifically, to assess the frequency of O2O take-out food consumption, respondents were queried, ‘On average, how frequently do you consume online ordered take-out food per week? (‘0~2 times’ = 1; ‘3~4 times’ = 2; ‘5~7 times’ = 3; ‘8~10 times’ = 4; ‘Over 10 times’ = 5)’ The survey also captured the frequency of specific conventional dietary behaviors that pertain to food consumption outside the O2O take-out sphere. This specifically encompassed the frequency of fruit and vegetable intake, which is associated with a lower risk of being overweight and obese [39, 60, 61]. Furthermore, the survey inquired about the frequency of fast food consumption, acknowledging its correlation with an elevated risk of overweight and obesity [9–14]. Fast food refers to ready-to-eat food that is quickly prepared and served using standardized methods. In this study, the primary difference between fast food consumption in regular diets and O2O take-out food consumption lies in whether the food is obtained through online ordering. The survey questions are ‘On average, how frequently do you consume fruits and vegetables in regular meals per week?’ and ‘On average, how frequently do you consume fast-food ordered in regular meals per week?’ respectively. The options are ‘0~2 times’ = 1; ‘3~4 times’ = 2; ‘5~7 times’ = 3; ‘8~10 times’ = 4; ‘Over 10 times’ = 5.

Consumers’ preferences toward specific food categories are likely to significantly shape their dietary behaviors [62], especially when the prevalence of online food delivery services simplifies their access to diverse food options. In the tools of assessing diet, an individual’s preference and inclination towards certain types of food is often used by Dietary screeners, and its effectiveness has been verified by previous research [63, 64]. Therefore, the survey also assessed O2O take-out food consumption behaviors by investigating preferences for ordering specific food categories. Participants were asked to express their agreement with statements such as ’I prefer to order Western-style fast food.’ This included examining preferences for ordering fast food through O2O take-out services, with a meticulous distinction between Western-style fast food and Chinese local fast food. Participants rated their agreement with statements about their preference for ordering from each category, using a five-point Likert scale from ’strongly disagree’ to ’strongly agree.’ Additionally, the survey documented preferences for ordering take-outs of fruits and vegetables. Other types of take-out foods addressed in preference-related inquiries encompassed barbecue, snacks, coffee, milk tea, and other beverages—items widely popular on O2O take-out food platforms in China [65].

### Dependent variable

This study uses a binary variable—whether or not one is overweight or obese—as the dependent variable, with Body Mass Index (BMI) as the criterion for determining overweight and obesity status. Participants were asked to self-report their height and weight. BMI was determined by dividing weight (in kilograms) by height squared (in meters) [66]. The overweight and obesity in this study were based on the guidelines set forth by China’s National Health and Medical Commission in 2013. Specifically, the normal range for adult BMI was defined as 18.5–23.9, while BMIs falling between 24–27.9 were considered overweight, and those above 28 were classified as obese [66].

### Control variables

The control variables of this study include energy expenditure and individual factors. The energy expenditure was measured in the form of physical activity, which is evaluated using the International Physical Activity Questionnaire (IPAQ) short form [67], widely applied in physical activity-related research [67, 68]. Since the survey was conducted in China, the Chinese version of the IPAQ was used. The reliability of this version has also been validated by previous studies [69]. A respondent was defined as physically ’active’, based on the ’Minimally Active’ standard in the IPAQ [67]. The ‘Minimally active’ is defined as a) three or more days of vigorous activity of at least 20 minutes per day, b) five or more days of moderate-intensity activity or walking of at least 30 minutes per day, or c) five or more days of any combination of walking, moderate-intensity, or vigorous-intensity activities achieving a minimum of at least 600 MET-min/week (MET is the weighting unit specified in IPAQ) [67]. Respondents who did not meet the standard were classified as ’inactive.’

The individual factors of the respondents included gender (male/female), age, marital status (married/single, divorced, widowed), educational level (college degree and above/below college degree), occupations (students/non-students), and income level. Respondents from Suzhou and Shanghai were also indicated and compared in the study.

### Statistical analysis

The O2O food options were not previously included in the FFQ and Dietary screeners. Therefore, the consistency and reliability of those new options need to be assessed. Cronbach’s alpha was employed to evaluate the internal reliability of the relevant survey questions [70–73]. The standardized Cronbach α coefficient of the items need to surpass the threshold of 0.6 and every Corrected Item-Total Correlation (CITCs) of the items need to surpass the threshold of 0.3 [70–74]. Additionally, Exploratory Factor Analysis (EFA) was used to determine the construct validity of the measurements [71, 72, 75, 76]. The Kaiser-Meyer-Olkin (KMO) value in the EFA need to surpass the threshold of 0.6 and all items demonstrated factor loading coefficients need to exceed 0.4 [71, 72, 75, 76].

Binary logistic regression models were developed to identify potentially significant variables associated with being overweight or obese. Given that those O2O and non-O2O take-out food consumption behaviors can either independently impact weight-related health outcomes or concurrently influence these outcomes when considered together, three binary logistic regression models were developed to comprehensively elucidate the intricate interplay between them. In addition to individual characteristics and energy expenditure variables, Model 1 includes only the non-O2O take-out food consumption variables as energy intake factors; Model 2 includes only the O2O take-out food consumption variables; Model 3 includes both sets of variables. To mitigate the risk of multicollinearity and ensure independent variable reliability, multicollinearity diagnostics were applied, confirming that the variables in the models do not exhibit high intercorrelations. The Events per Variable (EPV) for the three models will be calculated separately. If the EPV falls within the range of 5 to 15, it indicates that the study size is appropriate for analysis [77, 78]. All analyses were performed using IBM SPSS Statistics 27.

## Results

This study included a total of 716 valid respondents, with 400 participants from Shanghai and 316 from Suzhou.

### Descriptive statistics of participants

Table 1 presents the descriptive summary of the respondents, encompassing demographic characteristics, overweight and obesity status, physical activity status, and geographic locations.

**Table 1:** Descriptive statistics of the sample.

Items	Options	Count	Percentage (%)
**Age (Mean = 26.032, Median = 24, Std = 6.930)**	18–20	127	17.74
	21–25	327	45.67
	25–30	106	14.80
	31–40	120	16.76
	>40	36	5.03
**Gender**	Male	304	42.46
	Female	412	57.54
**Marital status**	Married	203	28.35
	Unmarried	513	71.65
**Education**	Up to high school degree	189	26.40
	Bachelor’s degree and more	527	73.60
**Occupation status**	Student	371	51.82
	Non-Student	345	48.18
**Personal monthly income (RMB)**	<2000	167	23.32
	2000–4000	194	27.09
	4000–6000	129	18.02
	6000–8000	128	17.88
	8000–10000	51	7.12
	10000–15000	32	4.47
	>15000	15	2.09
**Whether Overweight or obesity**	No	592	82.68
	Yes	124	17.31
**Physical activity status**	Active	477	66.62
	Inactive	239	33.38
**Location**	Shanghai	400	55.87
	Suzhou	316	44.13

The demographic breakdown of the survey participants included 42% male and 58% female respondents. Participants’ ages ranged from 18 to 58 years, with the mean age being 26 years and a standard deviation of 6.93. Notably, students comprised a considerable segment of the respondents at 52%, with a significant concentration of college students within the 21–25 years age bracket. Moreover, reported monthly income levels were modest, as over 68% of respondents earn under 6,000 RMB (approximately USD$820) per month. In terms of marital status, a majority (over 71%) were single, divorced, or widowed. The incidence of obesity or overweight status among the participants was 17.31%. Importantly, a notable 66.62% of respondents described themselves as being physically ’active’.

### Cronbach α reliability analysis and exploratory factor analysis (EFA) results

Following the application of the Cronbach α reliability test and the EFA for establishing validity, a set of five items demonstrated satisfactory levels of reliability and validity. Please refer to Tables 2 and 3 for a detailed analysis process.

**Table 2:** Reliability statistics of O2O take-out food consumption behaviors.

Items	The 1st Reliability Statistics			The 2nd Reliability Statistics		
	Corrected Item-Total Correlation (CITC)	Cronbach α if Item Deleted	Standardized Cronbach α	Corrected Item-Total Correlation (CITC)	Cronbach Alpha if Item Deleted	Standardized Cronbach α
**Frequency of O2O take-out food consumption**	0.356	0.553	0.599	0.365	0.688	0.699
**Preference for ordering Western fast-food**	0.348	0.561		0.406	0.651	
**Preference for ordering Chinese fast-food**	0.396	0.548		0.505	0.611	
**Preference for ordering vegetables**	0.407	0.542		0.459	0.627	
**Preference for ordering fruits**	0.436	0.534		0.516	0.602	
**Preference for ordering coffee or milk tea**	0.097	0.610				
**Preference for ordering snacks**	0.185	0.593				
**Preference for ordering barbecue food**	0.190	0.594				

**Table 3:** Validity analysis (EFA) of O2O take-out food consumption behaviors.

Items	Factor Loadings		Communalities
	Factor 1	Factor 2	
**Frequency of O2O take-out food consumption**	0.141	0.797	0.655
**Preference for ordering Western fast-food**	0.176	0.797	0.667
**Preference for ordering Chinese fast-food**	0.742	0.217	0.598
**Preference for ordering vegetables**	0.855	0.009	0.731
**Preference for ordering fruits**	0.717	0.281	0.593
**KMO**	0.732		
**p value of Bartlett’s Test of Sphericity**	p-value<0.05		

Those five items meeting reliability and validity test standards are as follows: (1) Frequency of take-out food consumption, (2) Preference for ordering Western fast food, (3) Preference for ordering Chinese fast food, (4) Preference for ordering vegetables, and (5) Preference for ordering fruits. Consequently, these items were employed in the binary logistic regression models for assessing the behavior of O2O take-out food consumption in this study. The descriptive statistics of these five items are shown in Table 4.

**Table 4:** Descriptive statistics of the items for assessing the behavior of O2O take-out food consumption.

Items	Options	Count	Percentage (%)
**Frequency of O2O take-out food consumption per week**	0–2 times	100	13.97
	3–4 times	179	25.00
	5–7 times	241	33.66
	8–10 times	120	16.76
	>10 times	76	10.61
**I Prefer to order Western fast-food take-outs**	Strongly disagrees	103	14.39
	Disagree	108	15.08
	I have no idea	153	21.37
	Agree	238	33.24
	Strongly agree	114	15.92
**I Prefer to order Chinese fast-food take-outs**	Strongly disagrees	55	7.68
	Disagree	282	39.39
	I have no idea	141	19.69
	Agree	127	17.74
	Strongly agree	111	15.50
**I Prefer to order vegetables take-outs**	Strongly disagrees	278	38.83
	Disagree	297	41.48
	I have no idea	96	13.41
	Agree	34	4.75
	Strongly agree	11	1.54
**I Prefer to order fruits take-outs**	Strongly disagrees	151	21.09
	Disagree	348	48.60
	I have no idea	162	22.63
	Agree	37	5.17
	Strongly agree	18	2.51

### Regression model results

The results of the binary logistic regression model are shown in Table 5, and the dependent variable of this model is whether an individual is overweight and obese or not (Yes = 1). The number of overweight and obesity samples is 124. The EPV of the three models are 12.4, 9.5 and 8.3 respectively, indicating that the study size is appropriate for analysis.

**Table 5:** Binary logistic regression model results.

		Model 1			Model 2			Model 3		
	Variables	OR	95%CI	p-value	OR	95%CI	p-value	OR	95%CI	p-value
**O2O take-out food consumption**	**Frequency of O2O take-out food consumption per week**				1.082	1.064 ~ 1.101	<0.001**	1.087	1.068 ~ 1.106	<0.001**
	**Preference for ordering Western fast-food take-outs**				1.062	1.041 ~ 1.085	<0.001**	1.071	1.046 ~ 1.095	<0.001**
	**Preference for ordering Chinese fast-food take-outs**				0.971	0.952 ~ 0.990	0.003**	0.990	0.969 ~ 1.012	0.384
	**Preference for ordering vegetables take-outs**				0.976	0.960 ~ 0.993	0.005**	0.996	0.977 ~ 1.015	0.651
	**Preference for ordering fruits take-outs**				0.988	0.969 ~ 1.007	0.212	0.993	0.973 ~ 1.013	0.480
**Non-O2O take-out food consumption**	**The frequency of fast-food consumption in regular meals per week**	1.016	1.004 ~ 1.028	0.011*				1.036	1.015~1.058	<0.001**
	**The frequency of fruits and vegetables in regular meals consumption per week**	0.972	0.957 ~ 0.987	0.032*				0.970	0.948 ~ 0.993	0.011*
**Energy expenditure**	**Physical activity status (Active = 1, Inactive = 0)**	0.158	0.100 ~ 0.249	<0.001**	0.116	0.062 ~ 0.216	<0.001**	0.099	0.051 ~ 0.193	<0.001**
**Individual factors**	**Gender (Female = 1, Male = 0)**	0.183	0.115 ~ 0.294	<0.001**	0.170	0.091 ~ 0.318	<0.001**	0.117	0.059 ~ 0.233	<0.001**
	**Age**	0.861	0.626 ~ 1.184	0.358	0.819	0.533 ~ 1.260	0.364	0.855	0.551 ~ 1.328	0.486
	**Marital status (Married = 1, Unmarried = 0)**	1.828	0.926 ~ 3.607	0.082	1.498	0.602 ~ 3.729	0.385	1.321	0.506 ~ 3.447	0.570
	**Education (Bachelor’s degree and more = 1, Up to high school degree = 0)**	0.797	0.521 ~ 1.219	0.295	0.601	0.331 ~ 1.089	0.093	0.611	0.333 ~ 1.119	0.111
	**Occupation status (Non-student = 1, Student = 0)**	0.981	0.653 ~ 1.472	0.925	0.876	0.520 ~ 1.473	0.617	1.023	0.593 ~ 1.766	0.935
	**Personal monthly income (RMB**	1.036	0.838 ~ 1.281	0.743	0.838	0.629 ~ 1.116	0.227	0.786	0.582 ~ 1.060	0.114
	**Location (Shanghai = 1, Suzhou = 0)**	0.537	0.286 ~ 1.006	0.052	0.248	0.111 ~ 0.558	<0.001**	0.264	0.115 ~ 0.607	<0.001**

Note:

* = p-value < 0.05

** = p-value < 0.01.

Dependent Variable: Whether overweight and obesity or not

In Model 1, including conventional dietary behaviors only, energy expenditure, and individual factors, the frequency of fast-food consumption per week and the frequency of fruit and vegetable consumption per week were significantly positively and negatively correlated with overweight and obesity, respectively, with odds ratios of 1.016 (95% CI: 1.004~1.028, p-value = 0.011) and 0.972 (95% CI: 0.957~0.987, p-value = 0.032).

In Model 2 with O2O take-out food behavior variables only, energy expenditure, and individual factors, four out of the five items related to O2O take-out food consumption behaviors exhibited statistical significance. Specifically, there was a significant positive correlation between the frequency of take-out food consumption and being overweight or obese, with an odds ratio of 1.082 (95% CI: 1.064~1.101, p-value<0.001). Similarly, a significant positive correlation was observed between the preference for ordering Western fast food and being overweight or obese, with an odds ratio of 1.062 (95% CI: 1.041~1.085, p-value<0.001). Conversely, the preference for ordering Chinese fast food and the preference for ordering vegetables displayed a significant negative correlation with overweight or obesity, with adds ratios of 0.971 (95% CI: 0.952~0.990, p-value = 0.003) and 0.976 (95% CI: 0.960~0.993, p-value = 0.005), respectively.

In Model 3, wherein both O2O take-out food consumption behaviors and conventional dietary behaviors were concurrently considered, subtle alterations in the outcomes are observed. Notably, the frequency of O2O take-out food consumption and the preference for O2O Western fast food maintained a statistically significant positive correlation with being overweight or obese, exhibiting odds ratios of 1.087 (95% CI: 1.068~1.106, p-value<0.001) and 1.071 (95% CI: 1.046~1.095, p-value<0.001), respectively. Additionally, the two conventional dietary behavior variables, namely the weekly frequency of fast-food consumption and the frequency of fruit and vegetable consumption, exhibited continued significant positive and negative correlations with being overweight and obese, respectively, with odds ratios of 1.036 (95% CI: 1.015~1.058, p-value<0.001) and 0.970 (95% CI: 0.948~0.993, p-value = 0.011). However, the remaining three variables of O2O take-out consumption behaviors did not demonstrate statistically significant relationships with being overweight or obese.

Moreover, the findings of all three models exhibited similarities concerning the individual factors. Specifically, the results indicated that men were more susceptible to being overweight or obese compared to women. At the same time, individuals who were physically active were less likely to be overweight and obese. Interestingly, residents in Suzhou were significantly more likely to be overweight and obese than their counterparts in Shanghai.

## Discussion

The study’s findings revealed a positive relationship between frequent use of O2O take-out food services and the risk of being overweight and obese. In particular, the frequency of O2O take-out food consumption was found to be significantly positively associated with being overweight and obese in the two regression models that included O2O take-out food consumption behavior variables. This finding is compatible with existing literature that positions food delivery services as a conduit for unhealthy eating habits [47]. This study highlights how the simple process of ordering, coupled with a plethora of high-calorie options available through these services, can foster overeating habits and a preference for processed foods over more nutritious alternatives. This trend is concerning, as it indicates a shift in eating habits towards convenience at the expense of nutritional value.

Furthermore, the findings corroborate the impact of Western-style fast food on obesity rates in Asian regions [30, 32, 35, 79, 80]. The preference for ordering Western fast-food take-outs and frequency of fast-food consumption outside of O2O take-out food orders were positively associated with being overweight and obese. This highlights a strong preference among consumers for ordering such food via O2O channels and in conventional settings. The study reveals a complex pattern of dietary habits linked to the allure of Western-style fast food. This underscores the broader societal need for interventions that target not just Western-style fast food availability and marketing but also focus on consumer education and the promotion of healthy eating alternatives.

In contrast, the consumption of local Chinese fast food depicts a more intricate scenario. Initial findings showed an unexpected inverse relationship with overweight and obesity, suggesting that, unlike Western-style fast food, local Chinese options might not uniformly lead to weight gain. However, this correlation weakens after accounting for fruit and vegetable intake. Such an adjustment suggests that individuals who frequent local Chinese fast-food establishments may generally adhere to a more balanced diet, replete with a variety of nutrient-rich foods, which may counterbalance the potential negative effects of fast food. This is consistent with the conclusion of a recent literature review, which suggests that a traditional Chinese diet, rich in vegetables, rice, and meat, correlates with reduced obesity risk^75^. This raises the hypothesis that local Chinese fast food may be lower in calories and fat, or its consumers might exhibit more nutritionally aware behaviors.

Additionally, it’s reasonable to consider that the lifestyle of consumers plays a significant role. It is plausible that the same cultural values influencing dietary choices promote a more active way of life. Regular physical activity, known to be a critical factor in maintaining a healthy weight [81–87], could very well be a prevalent characteristic among the consumers of local Chinese fast food, providing a buffer against the development of obesity.

These observations suggest a cultural or behavioral synergy between culinary preferences and lifestyle practices, meriting further inquiry into whether the nutritional composition of local Chinese and other non-Western fast foods inherently supports weight management or if the consumer base for these foods inherently adopts a more balanced diet.

After adjusting for dietary behaviors, this study uncovered that physical activity plays a protective role against being overweight and obese. This association aligns with the consensus of scientific evidence from previous research, which consistently shows that physical activity contributes to energy expenditure and is fundamental in achieving and maintaining a healthy weight [81–87]. The study’s findings reiterate the importance of regular physical exercise in the prevention and management of overweight and obesity by demonstrating its beneficial effects independently of diet.

Furthermore, an interesting geographic disparity in obesity rates was observed, with Shanghai residents showing lower obesity prevalence than those from Suzhou. This disparity may stem from a range of regional differences, from urban planning to socioeconomic dynamics and local health practices, indicating the potential influence of various environmental and lifestyle factors on public health.

This study is subject to several limitations. Firstly, the utilization of a cross-sectional research method hinders the establishment of a clear causal relationship. Secondly, this study was conducted online, which resulted in predominantly young participants. Previous studies from other countries have shown that younger individuals are more likely to use online food delivery services [49]. Given that 78% of our respondents were under 30 years of age, the overrepresentation of younger adults may limit the generalizability of our findings to the broader urban adult population. The data were collected through an online survey administered by a professional survey company, which predominantly attracted younger individuals more inclined to use online media and platforms for food ordering. This demographic concentration presents challenges in extrapolating the results to all urban residents. Therefore, it is essential to validate these findings through a longitudinal study specifically targeting this younger cohort, as they represent the primary demographic engaging with online food consumption technologies. Thirdly, this survey’s reliance on self-reported data such as height and weight, which may lead to limitations in data accuracy and precision. When feasible, data obtained through direct measurement using appropriate equipment would be more accurate. Finally, this study takes a relatively simple approach to the design of control variables, including individual factors such as socioeconomic status. In future research, if there is a specific need to focus on these aspects, more detailed classifications of variables such as educational level and occupation could be applied. Hence, the generalizability of the research findings merits further exploration.

## Conclusion

This study is one of the first studies investigating the effects of O2O take-out food on overweight and obesity and identifying a connection between the habitual use of such services and an increased propensity for these conditions. It underscores and adds to the growing body of evidence on the influence of Western-style fast food on weight gain and obesity. Simultaneously, it uncovers the intricate and less straightforward relationship between the intake of local Chinese fast food and weight status, suggesting a multifaceted interplay of factors. Collectively, this research enriches our comprehension of the relationships among O2O take-out food, dietary habits, cultural factors, physical activity levels, and their collective impact on overweight and obesity.
